# The intriguing strategies of *Tannerella forsythia's* host interaction

**DOI:** 10.3389/froh.2024.1434217

**Published:** 2024-05-30

**Authors:** Christina Schäffer, Oleh Andrukhov

**Affiliations:** ^1^ Department of Chemistry, Institute of Biochemistry, *NanoGlycobiology* Research Group, Universität für Bodenkultur Wien, Vienna, Austria; ^2^ Competence Center for Periodontal Research, University Clinic of Dentistry, Medical University of Vienna, Vienna, Austria

**Keywords:** host response, inflammatory mediators, protein *O*-glycosylation, S-layer, *Tannerella forsythia*, virulence factors

## Abstract

*Tannerella forsythia*, a member of the “red complex” bacteria implicated in severe periodontitis, employs various survival strategies and virulence factors to interact with the host. It thrives as a late colonizer in the oral biofilm, relying on its unique adaptation mechanisms for persistence. Essential to its survival are the type 9 protein secretion system and *O*-glycosylation of proteins, crucial for host interaction and immune evasion. Virulence factors of *T. forsythia*, including sialidase and proteases, facilitate its pathogenicity by degrading host glycoproteins and proteins, respectively. Moreover, cell surface glycoproteins like the S-layer and BspA modulate host responses and bacterial adherence, influencing colonization and tissue invasion. Outer membrane vesicles and lipopolysaccharides further induce inflammatory responses, contributing to periodontal tissue destruction. Interactions with specific host cell types, including epithelial cells, polymorphonuclear leukocytes macrophages, and mesenchymal stromal cells, highlight the multifaceted nature of *T. forsythia's* pathogenicity. Notably, it can invade epithelial cells and impair PMN function, promoting dysregulated inflammation and bacterial survival. Comparative studies with periodontitis-associated *Porphyromonas gingivalis* reveal differences in protease activity and immune modulation, suggesting distinct roles in disease progression. *T. forsythia's* potential to influence oral antimicrobial defense through protease-mediated degradation and interactions with other bacteria underscores its significance in periodontal disease pathogenesis. However, understanding *T. forsythia's* precise role in host-microbiome interactions and its classification as a keystone pathogen requires further investigation. Challenges in translating research data stem from the complexity of the oral microbiome and biofilm dynamics, necessitating comprehensive studies to elucidate its clinical relevance and therapeutic implications in periodontitis management.

## 1 Introduction

**Figure 1:**
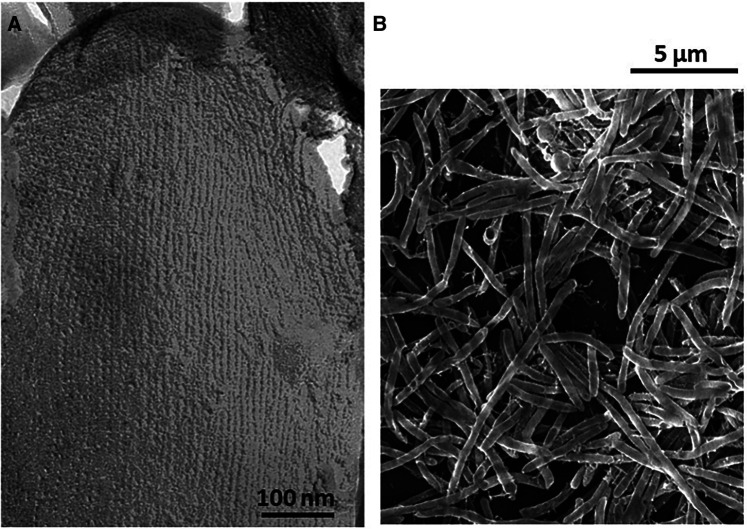
(**A**) Transmission electron microscopy of a freeze-etched and platinum carbon-shadowed *T. forsythia* cell showing the square S-layer lattice covering the entire cell surface. (**B**) Scanning electron microscopy image of rod-shaped *T. forsythia* cells grown on a hydroxyapatite disc.

Periodontitis is a highly prevalent inflammatory disease affecting the tooth-supporting tissues, impacting more than half of the adult population worldwide. Despite decades of intensive research, the precise etiological factors driving the initiation and progression of this disease remain elusive (1, 2). There is a consensus that periodontitis is triggered by a polymicrobial oral biofilm, with tissue destruction resulting from the host immune response to this biofilm. At the close of the last century, seminal work by Socransky and colleagues identified the “red complex,” comprising *Porphyromonas gingivalis*, *Treponema denticola*, and *Tannerella forsythia*, as associated with severe forms of periodontitis (2). Initially, these three Gram-negative anaerobes were considered as causative agents, but with the advent of sequencing technologies in oral microbiome research, it is now recognized that the etiology of periodontitis is far more complex (3). The progression of periodontitis is characterized by a shift from a symbiotic to a dysbiotic host-microbiome interaction in the oral cavity, driven by various risk factors and marked by an increase in bacterial biomass and alterations in the abundance of specific species (4–6). Notably, the relative abundance of red-complex bacteria significantly rises in subgingival plaque during periodontitis (7). While periodontitis is considered a polymicrobial infection with no sole pathogen, *P. gingivalis* is believed to play a distinctive role due to its ability to manipulate the host response, thereby tipping the balance from health to disease (5, 8). *P. gingivalis* has been extensively studied among periodontitis-associated bacteria, but research on other potential pathogens, particularly *T. forsythia* (Figure 1), remains limited. In this perspective paper, we describe important virulence factors of *T. forsythia* and explore the bacterium's potential to modulate the host response.

## 2 Survival strategies of *Tannerella forsythia*

*T. forsythia* and *P. gingivalis* are key members of the oral biofilm consortium, thriving as late colonizers (2). These bacteria are affiliated with the *Bacteroidetes* phylum and share several commonalities in cellular infrastructure, which are vital for their adaptation to the oral habitat and interaction with the host. This includes, e.g., the utilization of a type 9 protein secretion system (T9SS), a characteristic feature of *Bacteroidetes* members, for the export of specific virulence factors possessing a C-terminal structural targeting domain across the outer membrane (9–11). Additionally, they both have the capability to extensively *O*-glycosylate numerous proteins with a species-specific glycan (12–14). Deletion of the T9SS signal peptidase PorU in *T. forsythia* led to T9SS shutdown and reduced production of pro-inflammatory mediators by macrophages and gingival fibroblasts upon infection with the *ΔporU* deletion mutant compared to the *T. forsythia* parent strain (10).

*O*-glycosylation of cell surface proteins has been demonstrated to facilitate the persistence of *T. forsythia* in the host (15), likely through molecular mimicry of host sialic acid via the display of bacterial nonulosonic acids (16). However, complete immune evasion is not a viable strategy for either *T. forsythia* or *P. gingivalis*, as both rely on inflammation to obtain nutrients from tissue breakdown products (5).

In contrast to *P. gingivalis*, *T. forsythia* relies on other members of the biofilm consortium for survival due to its auxotrophy for the essential bacterial cell wall sugar *N*-acetylmuramic acid (MurNAc) (17). Consequently, the bacterium can only survive in the oral habitat by scavenging this compound from cell wall turnover products or decay of cohabiting bacteria (18), making it unique among oral bacteria. Notably, due to the absence of the MurAB enzymes involved in MurNAc biosynthesis, *T. forsythia* is resistant to the antibiotic fosfomycin (19).

## 3 Virulence factors of *T. forsythia*

### 3.1 Enzymes

#### 3.1.1 Sialidase

The surfaces of vertebrate cells are decorated with a dense array of sialoglycoproteins (20). The red-complex bacteria express sialidases to cleave terminal sialic acid from host glycoproteins, such as mucins, which can be used as a carbon source and/or modify host cell surfaces to enhance attachment (21). *T. forsythia* harbours a dedicated sialic acid utilization and scavenging (*nan*) operon (22), encoding a sialidase-NanH-which has been shown to play a crucial role in bacterial colonization by exposing sialic acid-hidden epitopes on epithelial cells (23). Furthermore, attachment and survival of *T. forsythia* during epithelial KB cell infection were found to be diminished in a mutant lacking the sialic acid transporter NanT (24). Notably, a NanS sialic acid-esterase from *T. forsythia* collaborates with a *P. gingivalis* sialidase to optimize *P. gingivalis'* acquisition of sialic acid, even from diacetylated sialic acid-containing substrates (25). This underscores the interdependencies among the red-complex bacteria for their interaction capability with the host.

#### 3.1.2 Proteases

Various proteases have been implicated in *T. forsythia's* pathogenicity, including, e.g., PrtH proteases which are associated with attachment loss (26), a trypsin-like cysteine protease exhibiting both arginine- and citrulline-specific activities (27), and secretory KLIKK proteases capable of targeting diverse protein substrates such as collagen, gelatine, elastin and casein (28). It is noteworthy that transcripts of KLIKK proteases were detected in almost all gingival crevicular fluid samples where *T. forsythia* was present. Specifically, miropin of *T. forsythia* deactivates a broad range of host proteases, including neutrophil elastases and cathepsin G (28), while karylisin inhibits complement activation at various stages (29). Additionally, mirolysin and karylisin have been shown to degrade and inactivate human cathelicidin (30, 31). These findings support the notion that these enzymes contribute to *T. forsythia's* pathogenicity through distinct mechanisms.

### 3.2 Cell surface proteins

#### 3.2.1 2D-crystalline cell surface (S-) layer and glycosylation

*T. forsythia* cells are covered by a 2D-crystalline array formed by the self-assembly of the S-layer proteins TfsA and TfsB, which are multiply modified by a nonasaccharide within the phylum-wide, three-amino acid *O*-glycosylation motif D(S/T)(A/I/l/M/T/V/S/C/G/F/N/E/Q/D/P) (12, 32, 33). The S-layer plays a crucial role in host interaction, as demonstrated by various studies utilizing a *T. forsythia* S-layer *ΔtfsAB* deletion mutant. Infection with the mutant led to an earlier onset of pro-inflammatory mediator production in macrophages and human gingival fibroblasts compared to the parent strain (34) and induced the production of monocyte chemoattractant protein and granulocyte-macrophage colony-stimulating factor by human oral epithelial cells (35). Furthermore, the *T. forsythia* S-layer deficient mutant exhibited significantly reduced adherence to human gingival epithelial cells (36).

Moreover, *O*-glycosylation of the S-layer proteins was found to modulate the interaction between *T. forsythia* and the host. Specifically, only the native nonasaccharide ensured the bacterium's persistence in the host, whereas mutants with truncated S-layer glycosylation, lacking the nonulosonic acid (37) (e.g., in a Δ*wecC* deletion mutant lacking a trisaccharide branch), elicited a robust Th17 response and reduced periodontal bone loss in mice compared to the *T. forsythia* wild-type bacterium (15). Immune priming with the Th17-biasing strain triggered a productive neutrophil response, effectively mitigating *P. gingivalis* persistence and associated alveolar bone loss in mice (38). Notably, initial evidence suggests that the glycosylated S-layer of *T. forsythia* is recognized by macrophage-inducible C-type lectin receptors (39), primarily expressed in myeloid cells, such as macrophages and neutrophils.

Regarding *T. forsythia's* biofilm lifestyle, using a subgingival, multispecies biofilm model, a selective role for the glycosylated S-layer was identified in positioning this bacterium within the biofilm, its co-localization with *P. gingivalis*, and the prevalence of *Campylobacter rectus* (40).

Thus, not only the presence of an S-layer but also its specific glycosylation pattern influences the host response to *T. forsythia*.

#### 3.2.2 Cell surface antigen BspA

*T. forsythia's* glycosylated cell surface antigen BspA triggers cytokine production in macrophages and dendritic cells via CD14 and TLR2-dependent mechanisms (41, 42); TLR2 activation is mediated through the sequence motif GC(S/T)GLXSIT (43). Animal studies have underscored the pivotal role of BspA in the cellular response to *T. forsythia*; mice infected by gavage with BspA-deficient *T. forsythia* displayed significantly reduced alveolar bone loss compared to those infected with the parent strain (43). Bone loss in infected mice was found to be linked to the activation of a Th2 response and notably decreased in animals lacking TLR2, highlighting a potential involvement of BspA in this process (42). Remarkably, the prevalence of the *T. forsythia* BspA genotype is markedly elevated in patients with chronic and aggressive periodontitis, suggesting a direct contribution of BspA to periodontal disease (44). Additionally, *T. forsythia* and BspA induce foam cell formation in THP-1 macrophages and promote the progression of atherosclerotic lesions in ApoE^(−/−)^ mice (45), indicating a potential impact of this virulence factor on systemic health.

### 3.3 Outer membrane vesicles

Outer membrane vesicles (OMVs) from *T. forsythia* induce the production of TNF-α and IL-8 in U937 macrophages, and IL-6, IL-8, and MCP-1 in human periodontal ligament mesenchymal stromal cells (hPDL-MSCs) in a concentration-dependent manner (46). The response of host cells to *T. forsythia* OMVs is comparable to or even stronger than that elicited by the whole bacterium (46) and is mediated through TLR2 activation (47). Monocytes and differentiated macrophages may phagocytose *T. forsythia's* OMVs, leading to the activation of a pro-inflammatory response (48). Interestingly, several cargo proteins within OMVs are virulence factors which are predictably *O*-glycosylated (46), supporting a potential link between glycosylation and virulence of *T. forsythia*.

### 3.4 Rough-type lipopolysaccharide

*T. forsythia* produces a rough-type lipopolysaccharide (LPS), which induces an inflammatory response in macrophages in a concentration-dependent manner (49, 50). Additionally, another study revealed that *T. forsythia* LPS stimulates cytokine production in whole blood from periodontitis patients. This response was further increased upon co-stimulation with *P. gingivalis* LPS or *T. denticola* LPS, resulting in notably elevated levels of IL-1β and TNF-α (51); this may contribute to the immunodestructive host response characteristic of periodontitis.

## 4 Cell-specific responses to *Tannerella forsythia*

### 4.1 Epithelial cells

The oral epithelium serves a crucial role in the protecting underlying structures against bacterial invasion, functioning as both a mechanical and immune barrier (52). *T. forsythia* has the ability to invade oral epithelial cells, a process facilitated by, e.g., the activation of phosphatidylinositol 3-kinase by the BspA protein (53, 54). Surface-associated and secreted BspA protein induces the production of IL-8 by these cells through a TLR2 dependent mechanism (55). Moreover, *T. forsythia* can attach to and invade the oral squamous cell carcinoma cell line H357, likely mediated by NanH sialidase (22). Evidence of *T. forsythia's* invasion of buccal epithelium has been demonstrated in an *in vivo* study (56). Interestingly, the bacterium has been found to have no effect on apoptosis in oral epithelial OKF6/hTERT-2 cells, in contrast to *P. gingivalis* and oral commensal *Streptococcus gordonii* and *Streptococcus sanguinis* (57). In the human oral keratinocyte cell line HOK-16B, *T. forsythia* induces the production of IL-24 mediated by reactive oxygen species (ROS) (58), as well as the activation of IL-1α production through nucleotide-binding oligomerization domain-like receptor protein 10 (59). Additionally, in human oral keratinocytes, *T. forsythia* stimulates the production of IL-1Rα, IL-8, and vascular endothelial growth factor (VEGF) (35). While *T. forsythia* slightly inhibited epithelial cell migration as measured in a scratch assay, this inhibition was markedly lower compared to that induced by *P. gingivalis* and *Prevotella nigrescens* (60).

### 4.2 Polymorphonuclear leukocytes

Polymorphonuclear leukocytes (PMNs), integral to the innate immune system, play a crucial role in eliminating invading pathogens through diverse mechanisms such as phagocytosis, ROS production, and neutrophil extracellular trap (NET) formation (61). PMNs represent the predominant immune cell type in the gingival crevice, and their continuous transmigration into the sulcus is essential for maintaining oral health (62). The ability to deactivate neutrophil function is considered a key strategy employed by periodontal pathogens to promote dysregulated inflammation (63, 64). Despite its importance, the specific interaction between *T. forsythia* and PMNs remains poorly investigated. A study demonstrated that PMNs can phagocytose non-opsonized *T. forsythia*, leading to ROS production (65). Additionally, *T. forsythia* has been shown to adhere to and invade dental follicle mesenchymal stem cells, resulting in diminished PMN chemotaxis, phagocytic activity, and NET formation induced by these cells (66). Such alterations in PMN function may contribute to enhanced survival of oral pathogens.

### 4.3 Macrophages

Macrophages, as phagocytic tissue-resident cells of the innate immune system, are ubiquitous in almost all tissues and undertake various functions, including phagocytosis, pathogen presentation, clearance of cellular debris, and regulation of tissue homeostasis (67–69). *T. forsythia*, along with its OMVs and LPS, elicits the production of diverse pro-inflammatory mediators, notably IL-1β, TNF-α, IL-6, and IL-8, in human U937 macrophages (10, 34, 46, 50, 70).

In murine macrophages, *T. forsythia* prompts the dose-dependent production of IL-6, MCP-1, and IL-23 (71). Furthermore, the co-infection of murine macrophages with *P. gingivalis* and *T. forsythia* synergistically enhances the production of IL-6, albeit not of other cytokines (71). One study has documented *T. forsythia*'s capability to induce macrophage apoptosis through the activation of caspase-1, concomitant with the release of danger signals, particularly fibronectin and heat-shock protein 60 (72).

### 4.4 Mesenchymal stromal cells

Mesenchymal stromal cells (MSCs) are widely distributed throughout dental tissues (73). These cells express pattern-recognition receptors and can be activated by various bacterial components (74). Similar to other MSCs, they possess immunomodulatory properties and can influence the activity of various cells. Therefore, dental MSCs are crucial for maintaining tissue homeostasis and play a significant role in the progression of periodontitis (75, 76). Several studies have demonstrated that *T. forsythia* induces the gene and protein expression of IL-6, IL-8, and MCP-1 in MSCs derived from the gingiva and periodontal ligament (10, 34, 70). A similar effect has been observed with *T. forsythia* LPS and OMVs (46, 50).

## 5 Discussion and perspectives

The current understanding of *T. forsythia's* pathogenicity mechanisms and host-interaction does not conclusively establish its capability to manipulate the host immune system and classify it as a keystone pathogen, similar to *P. gingivalis*, which is known to significantly influence host-microbiome interactions even when present at low abundance (8, 77). Various virulence factors of *P. gingivalis* are known to subvert the immune system. For instance, gingipain proteases inhibit local IL-8 accumulation, causing “local chemokine paralysis” that inhibits PMN migration and consequently reduces bacterial killing, contributing to bacterial overgrowth (78, 79). Gingipain proteases also impair T-cell function and tissue colonization through adhesion to epithelial cells (80). Additionally, *P. gingivalis* affects the host immune response by manipulating complement and TLR2 signaling (81). Further research into *T. forsythia's* virulence factors and their mode of action is necessary to understand if and how this bacterium can manipulate the host immune system.

Our recent studies have shown contrasting effects of *P. gingivalis* and *T. forsythia* wild-type bacteria on gene expression and corresponding content of various pro-inflammatory mediators in human macrophages and gingival and periodontal-ligament derived MSCs. While infection with *T. forsythia* induced both gene expression and protein content, infection with *P. gingivalis* resulted in increased gene expression but low levels of cytokines (10, 82). This observation suggests that *P. gingivalis* proteases have a greater ability to degrade host proteins than *T. forsythia* enzymes. Animal studies further support this, demonstrating that unlike *P. gingivalis*, subcutaneous infection of mice with *T. forsythia* does not inhibit neutrophil migration to the infected site (83).

*T. forsythia* may potentially affect oral antimicrobial defense through its proteases. Miropin, for instance, degrades neutrophil elastases and cathepsin G, impairing antibacterial neutrophil function (84). Moreover, *T. forsythia*, along with other red-complex bacteria, degrades cathelicidin, an essential antimicrobial peptide in the oral cavity (85). Additionally, *T. forsythia* inhibits complement activation at several stages through the metalloproteinase karylisin (29). Interestingly, a clinical study has shown a negative correlation between the prevalence and levels of *T. forsythia* in subgingival plaque and PMN activities such as phagocytosis and superoxide anion production (86).

There are indications that *T. forsythia* may alter the host response to other bacteria. Recent studies suggest that *T. forsythia* may scavenge NOD-2 ligands secreted by *F. nucleatum*, resulting in diminished activation of oral epithelial cells (87). *T. forsythia* also inhibits the invasion *of P. gingivalis* into oral epithelial cells (88) and synergistically augments alveolar bone loss with *F. nucleatum* in an experimental periodontitis model in mice (89). However, periodontitis models in mice primarily use ligature and infection with *P. gingivalis*, with limited studies using *T. forsythia*.

Translating available research data on *T. forsythia's* interaction with the host immune system is challenging. Most knowledge is based on *in-vitro* and sometimes mice studies using a model of mono-infection with *T. forsythia*, while the subgingival plaque contains numerous microorganisms, and host responses involve an orchestrated response of various cell types that is difficult to mimic in the laboratory.

Moreover, it's widely acknowledged that bacterial virulence is heavily influenced by ecological conditions and can be significantly altered within biofilms. For instance, the growth and virulence of various strains of *P. gingivalis* are contingent upon factors such as temperature, pH, and iron availability (90–92). While ecological conditions in the subgingival pocket may drive the selection of specific *T. forsythia* strains, data in this regard are limited.

Recent research has uncovered differences in the activation of the chemokine protein IP-10 by THP-1 macrophages between laboratory-adapted *T. forsythia* ATCC 43037 and clinical isolates (93). Notably, one clinical isolate elicited a notably higher response than *T. forsythia* ATCC 43037 (93). Additionally, another study highlighted a substantial increase in the prevalence of *T. forsythia bspA* and *prtH* genotypes in periodontitis patients compared to healthy individuals. While the underlying reason for this observation remains unclear, it is conceivable that factors such as increased GCF flow and alterations in its composition in periodontitis might drive the selection of specific *T. forsythia* strains or influence the gene expression and virulence of this bacterium (94).

**Figure 2:**
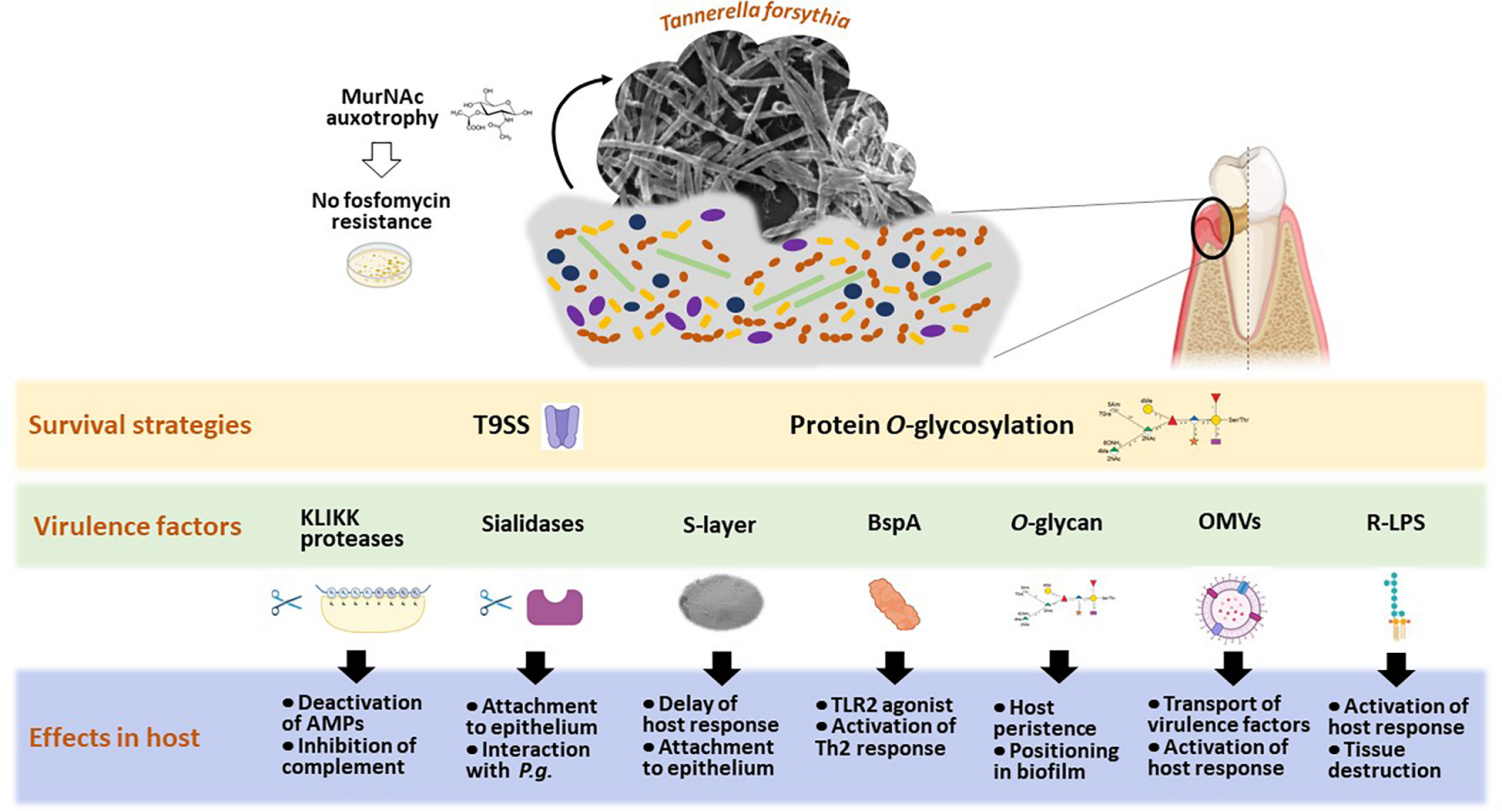
Scheme of *Tannerella forsythia's* host interaction strategies, illustrating the most investigated means of survival and known virulence factors leading to measurable effects in diverse host cells. Created with Biorender.

In summary, *T. forsythia* expresses several unique virulence factors that affect host defense and immune response (Figure 2). However, it remains unclear if *T. forsythia* has the potential to manipulate the host response, influence host-microbiome interactions, and promote a dysbiotic state. New studies focusing on the modification of oral biofilm by *T. forsythia*, the activity of *T. forsythia* proteases, and their clinical relevance, as well as their effects on the microbial community-host interaction, are necessary.

## Data Availability

The original contributions presented in the study are included in the article, further inquiries can be directed to the corresponding author.
